# Etiological Profile of Hospitalized Severe Acute Respiratory Infection (SARI) Patients During the COVID-19 Pandemic: A Cross-Sectional Study

**DOI:** 10.7759/cureus.80889

**Published:** 2025-03-20

**Authors:** Anjali Zoting, Swati Bhise, Priyanka Mategadikar, Pravin Deshmukh, Sunanda Shrikhande

**Affiliations:** 1 Microbiology, All India Institute of Medical Sciences, Raipur, Raipur, IND; 2 Microbiology, Indira Gandhi Government Medical College, Nagpur, IND; 3 Microbiology, Government Medical College, Nagpur, Nagpur, IND; 4 Microscopy, Government Medical College, Nagpur, Nagpur, IND

**Keywords:** covid-19, lower respiratory tract infections, rt-pcr, sari, sars-cov-2

## Abstract

Background

Lower respiratory infections remain one of the top global causes of death. The application of molecular diagnostic methods (e.g., reverse transcription-polymerase chain reaction {RT-PCR} panels) for the diagnosis of lower respiratory tract infections (LRTIs) improves the understanding of respiratory pathogen epidemiology of these diseases and helps in the early detection of causative agents and formulating infection control measures and management.

Methods

In this study, consecutive nasopharyngeal/oropharyngeal swab, sputum, tracheal aspirate, and bronchoalveolar lavage (BAL) samples collected from patients having respiratory symptoms were tested using RT-PCR.

Results

Out of 372 samples, respiratory pathogens were detected in 245 (65.86%) cases. The total number of viral isolates detected in this study was 235, including the viral co-infections and viral and bacterial mixed infections, out of which SARS-CoV-2 was most common (115, 48.94%), followed by influenza A(H1N1)pdm09 (82, 34.89%), rhinovirus (17, 7.23%), adenovirus (nine, 3.83%), influenza A (eight, 3.40%), and influenza B (four, 1.70%).

Conclusion

The rapid detection of respiratory pathogens through molecular methods can help with targeted antiviral treatment, limit the use of antibiotics, and help in knowing the burden of the disease.

## Introduction

Acute respiratory infections continue to remain a challenge to healthcare in the modern day.

Although they are most often self-limited and confined to the upper respiratory tract, they lead to a substantial number of upper or lower respiratory tract complications.

The World Health Organization (WHO) estimates that acute respiratory infections account for 1.9-2.2 million childhood deaths annually, with 70% occurring in Africa and Southeast Asia [1]. The causative agents include mainly viruses, up to 60% (e.g., respiratory syncytial virus {RSV}, influenza A, rhinovirus, adenovirus); bacteria (e.g., *Streptococcus pneumoniae*, *Mycoplasma pneumoniae*, and *Staphylococcus aureus*); and fungi (e.g., *Pneumocystis jirovecii*) [1,2].

The viral etiologies are hard to determine, and the unjustified use of antibiotics not only is expensive but also leads to a rapid increase in antimicrobial resistance to bacteria causing respiratory infections, so it is necessary to establish a diagnosis before the right treatment. The study was conducted with the objective of detecting viruses causing lower respiratory tract infection (LRTI) in the general population using multiple diagnostic tools.

## Materials and methods

This study was a hospital-based cross-sectional study held from January 2021 to December 2022 at a tertiary care center, which included a total number of 372 patients from all age groups with symptoms of severe acute respiratory infections (SARI; according to the WHO, an acute respiratory infection with a history of fever or measured fever of ≥38°C and cough, with onset within the last 10 days and which requires hospitalization). The Board of Research Studies (BORS) of Government Medical College, Nagpur, issued approval MUHS/Medical/MUHS-034040/2019. Unwilling patients and pregnant women were not included. Sputum and bronchoalveolar lavage (BAL) samples from patients clinically suspected of having respiratory tract infection were transported to the State Viral Diagnosis and Research Laboratory under a strict cold chain in sterile containers. Nasopharyngeal/oropharyngeal swabs were collected in (HiMedia's HiViral, Mumbai, India) viral transport medium (VTM) (3 mL). All samples were stored at -80°C until RNA extraction. RNA extraction was performed using MagRNA-II Viral RNA Extraction Kit by Genes2ME (Gurgaon, India) as per the instructions given in the kit insert.

Detection of respiratory pathogens by reverse transcription-polymerase chain reaction (RT-PCR)

The COVIDsure Multiplex Realtime RT-PCR Kit (Trivitron Healthcare, Chennai, India) was used for the in vitro detection of SARS-CoV-2 in respiratory specimens. The real-time PCR was done in a single-tube PCR system, and instructions were followed as per the kit insert. The quality of specimen collection was checked by the presence of internal control. Positive and negative controls were included in all the test samples. The presence of viral targets as mentioned for specific genes (*Orf1ab* gene, FAM; *E* gene, HEX; and *RPP30* gene, ROX) was used for determining the results along with Ct values. The cutoff criteria for the Ct value is per kit instructions. The RealStar Influenza Screen and Type RT-PCR Kit 4.0, a reagent system based on real-time PCR technology, was used for the qualitative detection and differentiation of influenza A virus, influenza B virus, and influenza A(H1N1)pdm09 virus-specific RNA. BIOFIRE FILMARRAY Pneumonia Panel (bioMerieux, Marcy-l'Étoile, France), a simplified molecular testing tool, was used, which works through a completely automated protocol beginning with integrated sample preparation and ending with the automated analysis of results. The following are the pneumonia panel tested in BIOFIRE FILMARRAY: bacteria: *Acinetobacter calcoaceticus-baumannii* complex, *Enterobacter cloacae*, *Escherichia coli*, *Haemophilus influenzae*, *Klebsiella aerogenes*, *Klebsiella oxytoca*, *Klebsiella pneumoniae* group, *Moraxella catarrhalis*, *Proteus* spp., *Pseudomonas aeruginosa*, *Serratia marcescens*, *Staphylococcus aureus*, *Streptococcus agalactiae*, *Streptococcus pneumoniae*, and *Streptococcus pyogenes*; atypical bacteria: *Legionella pneumophila*, *Mycoplasma pneumoniae*, and *Chlamydia pneumoniae*; and viruses: adenovirus, coronavirus, human metapneumovirus, human rhinovirus/enterovirus, influenza A virus, influenza B virus, parainfluenza virus, and respiratory syncytial virus.

## Results

**Figure 1:**
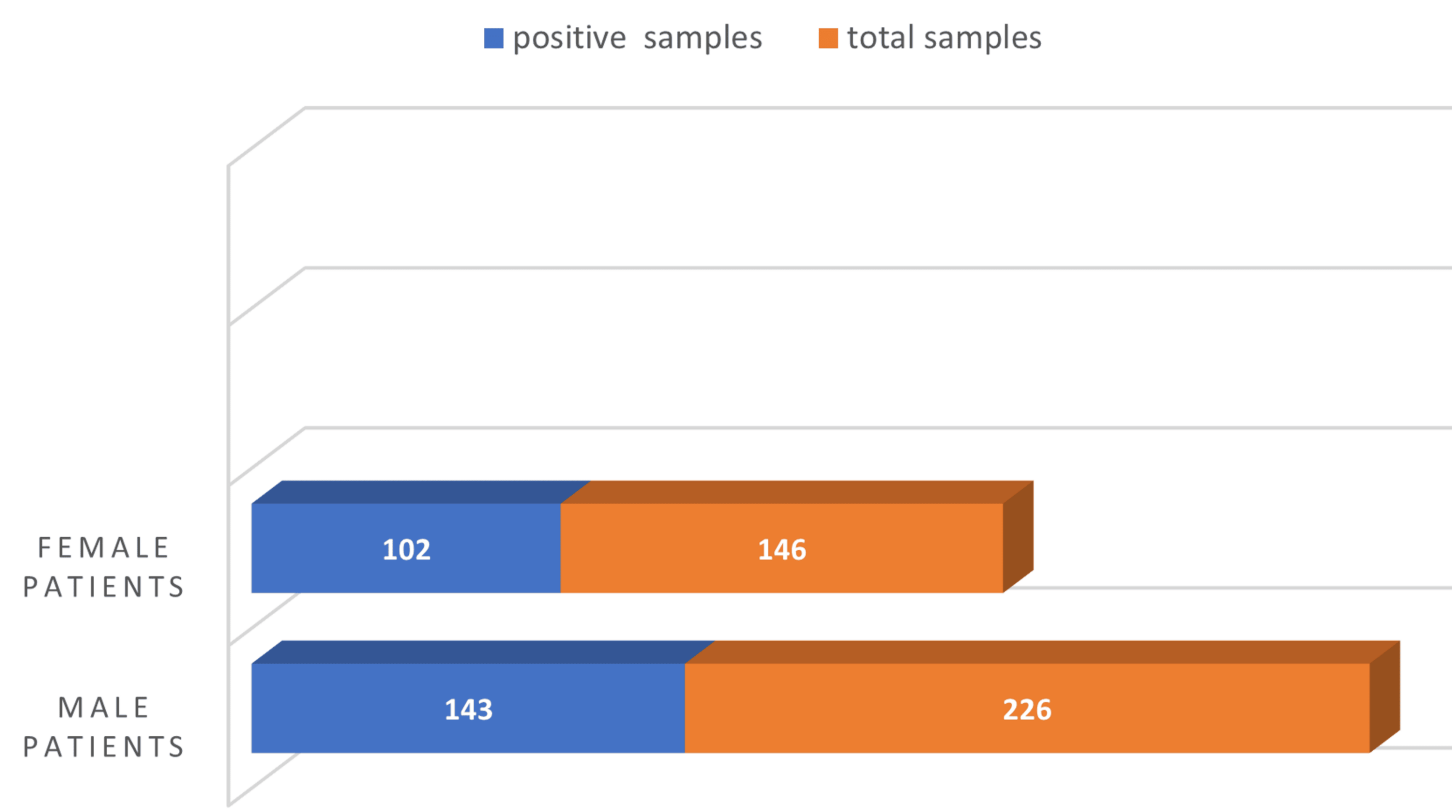
Incidence of respiratory pathogens in patients with lower respiratory tract infection

Out of 372 samples collected from patients with respiratory symptoms, the majority of the samples were from male patients (226, 60.75%), and the rest were from female patients (146, 39.25%). Respiratory pathogens were detected in 245/372 (65.86%) cases. Out of these, 143 were from men and 102 from women. Percentage positivity was higher in women (102/146, 69.86%) than in men (143/226, 63.27%) as shown in Figure 1. The difference was not found to be statistically significant (p-value = 0.192).

**Figure 2:**
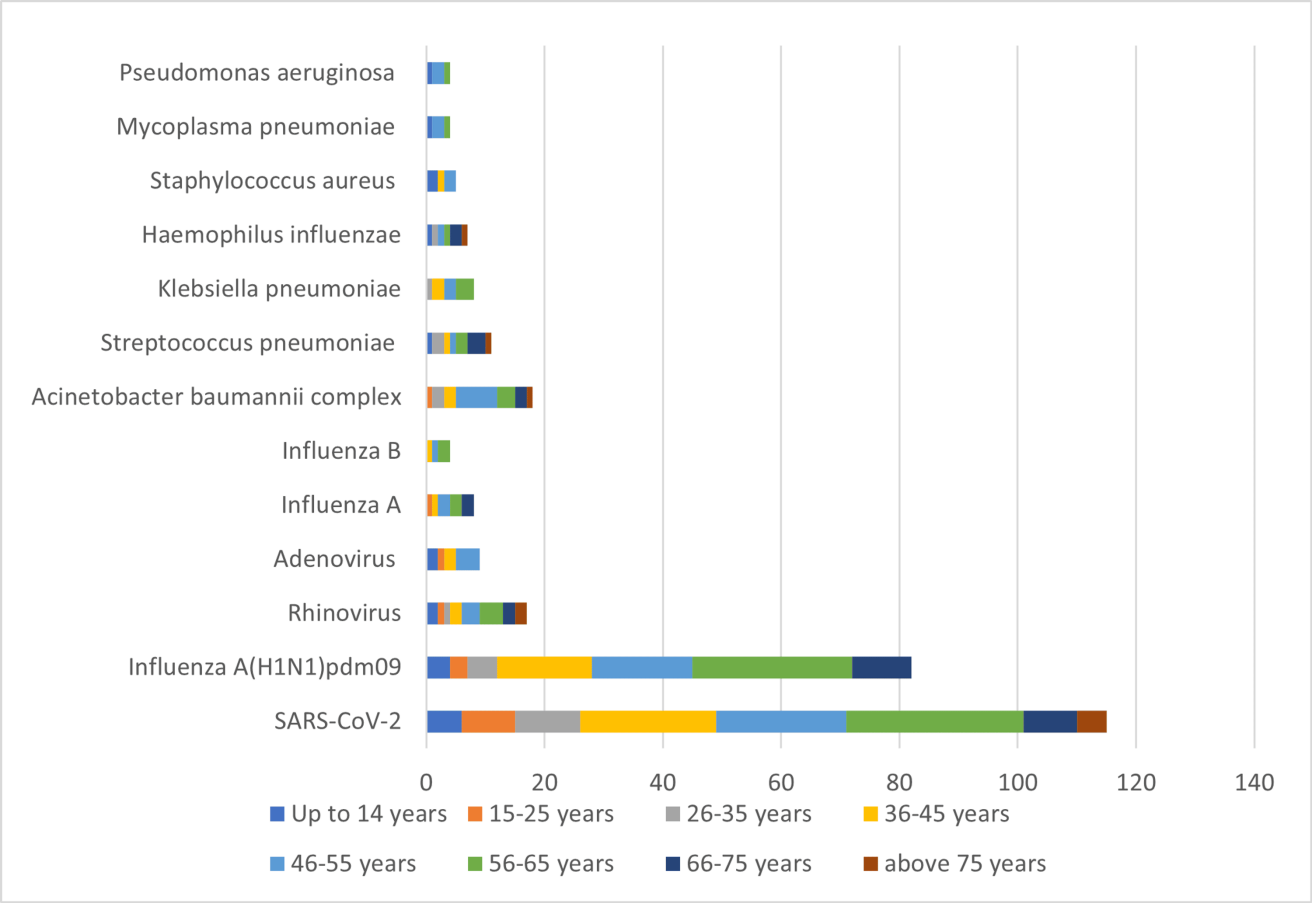
Age-wise distribution of various pathogens observed in the study

The majority of patients belonged to the 36-65-year age category. Percentage positivity was found higher in 56-65 years (79.01%), followed by 46-55 years (72.85%). The study population belonging to 26-35 years showed the least occurrence at 45.83%. For the study population, the age difference was statistically significant (p-value = 0.012). The age-wise distribution of various pathogens observed in the study is depicted in Figure 2.

Of the 372 respiratory samples collected, the majority were nasopharyngeal and oropharyngeal swabs (215, 57.80%). The least number of samples was BAL (12, 3.22%). It was determined that the p-value (0.407) was statistically insignificant. The majority of the samples that tested positive for a respiratory pathogen (n = 245) had a single virus infection (80.41%, 197/245). In 9.38% (23/245) of the cases, a mixed viral and bacterial infection was identified. Seven out of 245 samples, or about 2.86%, tested positive for two or more viruses. Of the patients, 7.34% (18/245) had bacterial infection.

The majority of samples tested positive for a single virus. The most frequently detected virus in the study population was SARS-CoV-2 (102, 50%), followed by influenza A(H1N1)pdm09 (67, 32.84%). Rhinovirus (5.88%), influenza A (3.92%), influenza B (1.96%), and adenovirus (1.96%) were among the other viruses identified in the study. SARS-CoV-2 and influenza A(H1N1)pdm09 co-infection was detected in four (1.96%) cases. In two (0.98%) cases, influenza A(H1N1)pdm09 and adenovirus co-infection was present. Co-infection involving SARS-CoV-2, influenza A(H1N1)pdm09, and rhinovirus was found in one case (0.49%).

*Acinetobacter calcoaceticus-baumannii* complex (22.22%) was found commonly among 18/245 (7.34%) samples that tested positive for bacterial infection. The co-infection of *Klebsiella pneumoniae* (two, 11.11%) and *Staphylococcus aureus* (one, 5.55%) was seen with *Acinetobacter calcoaceticus-baumannii* complex. *Streptococcus pneumoniae* (three, 16.66%), *Mycoplasma pneumoniae* (two, 11.11%), and *Haemophilus influenzae* (two, 11.11%) were among the other bacteria found in the study.

There were 23/245 (9.38%) samples that tested positive for bacterial and viral infections combined. Coronavirus and *Acinetobacter calcoaceticus-baumannii* combination was detected the most commonly (21.73%).

In total, 235 viral isolates were detected in the study, including viral co-infections and viral and bacterial mixed infections. SARS-CoV-2 was the most common, with 115 cases (48.94%), followed by influenza A(H1N1)pdm09 (82, 34.89%), rhinovirus (17, 7.23%) adenovirus (nine, 3.83%), influenza A (eight, 3.40%), and influenza B (four, 1.70%).

**Figure 3:**
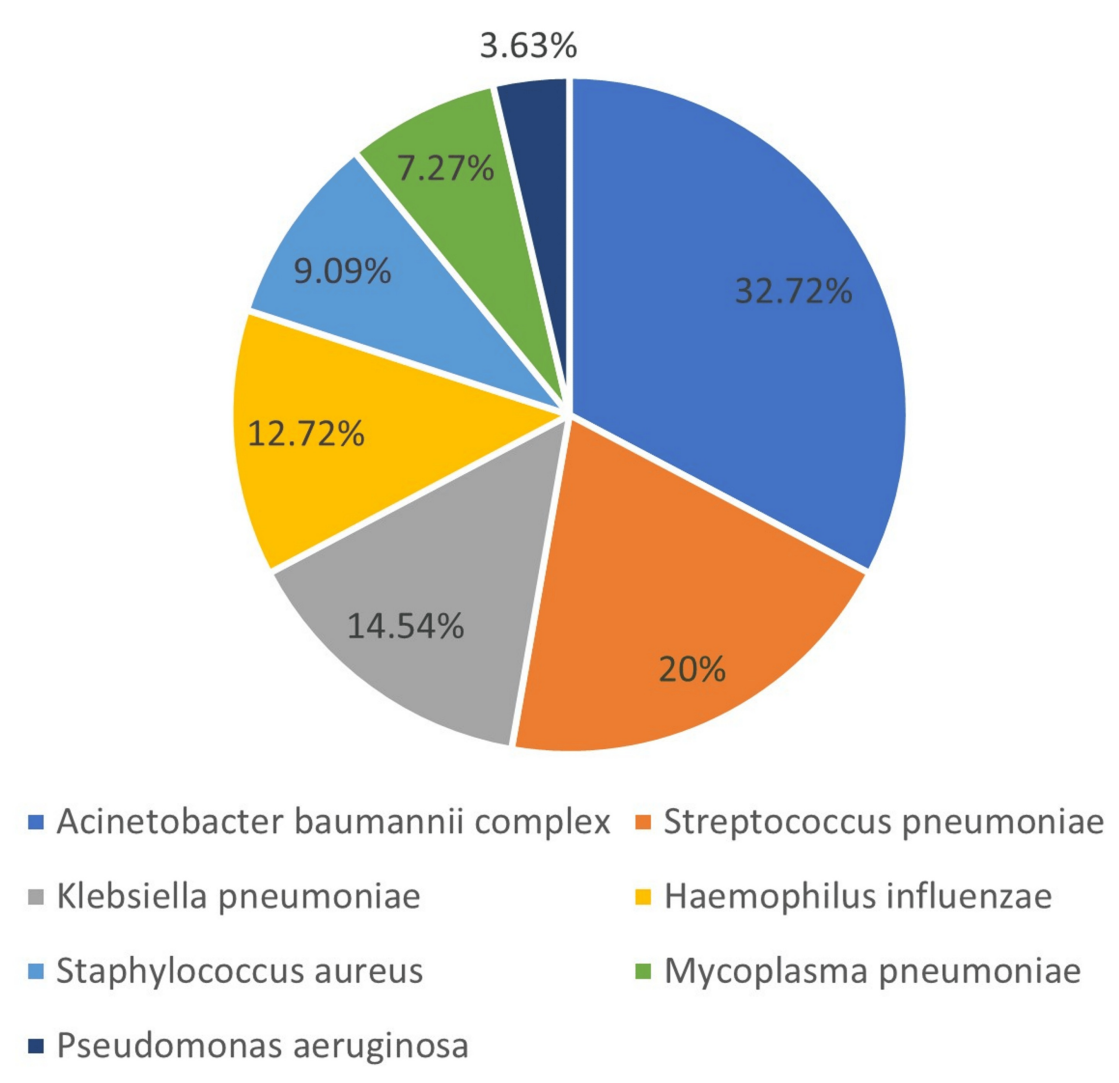
Profile of bacterial isolates detected in the study

In total, 55 bacterial isolates were found in the investigation. *Acinetobacter calcoaceticus-baumannii* complex made up 18 of these, or (32.72%) of the total, followed by *Streptococcus pneumoniae* (11, 20%), *Klebsiella pneumoniae* (eight, 14.54%), *Haemophilus influenzae *(seven, 12.72%), *Staphylococcus* *aureus* (five, 9.09%), *Mycoplasma pneumoniae* (four, 7.27%), and *Pseudomonas aeruginosa* (two, 3.63%) (Figure 3).

## Discussion

The present study was conducted in central India in a tertiary care center from January 2021 to December 2022. A total number of 372 consecutive samples were collected from patients of all age groups, suffering from respiratory tract infections. Of these, 226 (60.75%) samples were from male patients and 146 (39.25%) from female patients. The male-to-female ratio was 1.54:1. The Majority of patients belonged to the 36-65-year age category. Percentage positivity was found higher in 56-65 years (79.01%), followed by 46-55 years (72.85%). In a study by Prasetyo et al., the mean age of the study patients was 56.1 years with male preponderance [3]. In a study by Njouom et al., male preponderance was observed (50.1%) over women (48.8%) [4]. However, in this study, percentage positivity in women was found more than in men (p-value = 0.192). A study by Kumar et al. stated that the incidence of LRTI among the age group of 60-64 years was maximum [5]. In a study by Panda et al., LRTI was found more in the 51-60-year age group (n = 24), and men were found more at risk than women [6]. The incidence of LRTI increased with age among both men and women. While older patients were disproportionately impacted by SARS-CoV-2 infection, a significant percentage of younger patients needed both hospitalization and ICU treatment because of COVID-19, supporting the finding that this disease is not always mild in young individuals [7].

The majority of samples collected were nasopharyngeal and oropharyngeal swabs (57.80%). For the detection of the agents that cause lower respiratory tract infections (LRTIs), bronchoalveolar lavage (BAL) has been regarded as the gold standard, particularly in patients with complex or severe airway diseases, such as children with chronic lung diseases like cystic fibrosis or recurrent LRTIs [8]. However, guidelines advised a relative contraindication to bronchoscopy following the COVID-19 pandemic declaration since performing this aerosol-generating technique on a patient who has a known or suspected COVID-19 infection may raise the risk of infection for medical personnel [9]. Hence, samples (BAL) were the least collected samples (3.22%) in the present study (p-value = 0.407).

Respiratory pathogens were detected in 245 (65.86%) cases in the present study. Potential pathogens were identified in patients (69%) with LRTI by Creer et al. [10]. The identification rate was 81.03% for viruses and 18.97% for bacteria. A study by Sapra et al., similar to our study, revealed that 89.2% of the cases had just one pathogen present, 78.9% of the cases had SARS-CoV-2 positivity, and 10.8% had dual infection [11].

The most typical initial sign of influenza is thought to be fever. Numerous studies have demonstrated that patients with fever and cough within 48 hours after the onset of symptoms are likely to have influenza while it is circulating in the community [12]. Similar to this, cases of local transmission were reported shortly after imported cases were discovered in the affected countries in late December 2019 when a novel pathogen, SARS-CoV-2, first appeared in mainland China and the outbreak of acute respiratory disease (ARD) caused by this novel coronavirus (nCoV) turned into a global pandemic [13,14]. All patients with febrile pneumonia were to be isolated as part of admission techniques employed during Severe acute respiratory syndrome (SARS) outbreaks, and individuals based on this segregating factor were then submitted to RT-PCR testing for SARS-CoV-2 [15]. The presence of SARS-CoV-2 has received more attention during the pandemic phase, but co-infection in SARI/influenza-like illness (ILI) patients with or without SARS-CoV-2 has been overlooked. This explains why SARS-CoV-2 was more frequently detected in the current study (48.94%).

Sapra et al. found out that among non-COVID-19 SARI patients, human rhinovirus (48.5%), *Mycoplasma pneumoniae* (21.2%), influenza A virus (9.1%), influenza A/H1N1 (6.1%), and one (3%) each of HKU1, OC43, HPIV-1, HRSV, and HBoV were detected [11]. Influenza A(H1N1)pdm09 was the most prevalent among non-COVID-19 SARI patients in our study (34.89%). Since 2009, an outbreak of H1N1 of swine origin has replaced many seasonal influenza A viruses. Similar observations were made by Malhotra et al. [16] and Blyth et al. [17].

Ampuero et al. observed adenovirus in 6.2% of adult patients [18]. Kalimuddin et al. observed adenovirus in 6.3% of patients [19]. In this study, adenovirus was observed in a lesser number of patients (nine, 3.82%) than in the above two studies.

In this study, influenza A was detected in 3.40% of cases; it may be non-swine flu H1N1, H3N2, or another form. The RealStar Influenza Screen & Type RT-PCR Kit only detects swine flu as an influenza A subtype. Influenza A viruses regularly create epidemics with significant rates of morbidity and excess mortality as a direct result [20].

From 2001 to 2002 through the 2010-2011 seasons (excluding the 2009-2010 pandemic period), influenza B has been isolated in up to 44% of laboratory samples in the United States and in up to 60% of samples in Europe, with seasonal averages of 24% and 23%, respectively [21]. The age groups most susceptible to influenza B and the respective contributions of the two type B lineages (B/Victoria and B/Yamagata) to the illness burden are both poorly understood [22]. The co-circulation of Victoria- and Yamagata-like strains was discovered in Eastern India by Roy et al. [23]. Four cases (1.70%) were seen in this study. This indicates that influenza B should be included in the testing panel for respiratory pathogens.

A total of 204 out of 372 patients (54.83%) yielded single or multiple viruses without bacteria, where the use of high-end antibiotics could have been avoided.

In this study, among bacterial etiology, *Acinetobacter calcoaceticus-baumannii* complex (18, 32.72%) was most common, followed by *Streptococcus pneumoniae* (11, 20%), *Klebsiella pneumoniae* (eight, 14.54%), *Hemophilus influenzae* (seven, 12.72%), *Staphylococcus aureus* (five, 9.09%), *Mycoplasma pneumoniae* (four, 7.27%), and *Pseudomonas aeruginosa* (two, 3.63%). A study conducted by Yang et al. (2021) investigated the frequency and characteristics of respiratory co-infections in COVID-19 patients in the ICU; they detected that *A. baumannii* and *S. aureus* were more frequently identified during late ICU admission [24]. These infections increased in COVID-19 patients due to disease severity and have been associated with prolonged hospitalization and associated immune dysfunction [25].

In 23 (9.41%) of the cases, the virus and bacteria combination was present; most often identified among them (21.73%) was the coronavirus and *Acinetobacter calcoaceticus-baumannii* combination. Previous studies have shown that viral infection may pave the way for bacterial infection [26]. Mixed infections have been identified in a variable range of 16%-38% of cases of infection [27]. Some studies from Singapore have shown that only eight (1.8%) out of 431 COVID-19-positive patients had co-infection [28]. A study by Wang et al., from China, has shown that 5.8% of SARS-CoV-2-infected patients and 18.4% of patients without COVID-19 had co-infection with other respiratory pathogens [29]. In a study by Sapra et al., four patients had dual infection: Two patients had human rhinovirus and enterovirus infection, one had *Mycoplasma pneumoniae* with H1N1, and one had *Mycoplasma pneumoniae* with influenza A virus (other than H1N1) [11].

There are some limitations to the study; that is, it was conducted in a specific timeframe during the COVID-19 pandemic, which may have led to the underestimation of other infections, and it is not generalizable to other populations such as the pediatric age group or immunocompromised patients. This study had resource constraints, which may have affected the detection methods; subtypes of viral isolates could not be detected.

## Conclusions

SARS-CoV-2 and other stated viruses observed in this study affect symptoms resembling pneumonia. In primary care, it is challenging to distinguish between viral and bacterial etiologies. It is necessary to conduct more research to provide a customized method for an individualized risk assessment and then plan the treatment. This should include the characterization of different clinical aspects and symptoms and testing for viral etiology, which is often ignored. It will help in the judicious use of antibiotics, planning the economic burden efficiently, and formulating preventive measures.
